# Quality of Life After Hip Fracture Surgery in the Elderly: A Cross-Sectional Study

**DOI:** 10.7759/cureus.52631

**Published:** 2024-01-20

**Authors:** Bassem I Haddad, Mohammad Abu Ali, Oubaida Alashkar, Dana Jamos, Ibrahim Alnaser, Osama Qambar, Razan Aburumman, Dergam Altarawneh, Abdulrahman M Karam, Mohammad A Alshrouf

**Affiliations:** 1 Department of Orthopedics, Jordan University Hospital, Amman, JOR; 2 General Medicine, The University of Jordan, Amman, JOR; 3 Internal Medicine, Jordan University Hospital, Amman, JOR; 4 School of Medicine, The University of Jordan, Amman, JOR

**Keywords:** femur neck fractures, intertrochanteric fractures, hip fracture, qol, quality of life

## Abstract

Purpose

Hip fractures are common and serious injuries as they lead to high mortality and morbidity and have a significant effect on patients' lives. Additionally, these injuries have substantial socioeconomic consequences for patients' quality of life, their families, and healthcare systems. The aim of this study is to assess the quality of life (QoL) in patients after hip fracture surgery.

Methods

This study involved a cross-sectional survey between February 2016 and December 2019, with a sample of 199 patients who suffered a hip fracture and were treated at a tertiary care teaching hospital. The participants completed the EuroQol 5-Dimensions 5-Levels (EQ-5D-5L) questionnaire. Pearson's chi-squared test, independent sample t-test, and Pearson's correlation coefficient (r) were used in the analysis.

Results

We found that there is a statistically significant association between age and having problems with mobility (p=0.023), self-care (p<0.001), and usual activity (p=0.029). In addition, increased age was significantly associated with decreased EuroQol Visual Analog Scale (EQ-VAS) scores (r=-0.213, p=0.003). We also found a statistically significant association between gender and self-care, as males were more likely to report having problems with self-care when compared to females (OR: 3.63; CI 95%: 1.77-7.44; p<0.001).

Conclusion

Mobility, self-care, and usual activity were the most significantly affected quality of life measures and were more apparent in older age groups. Patients should be educated about the possibility of a decline in their QoL and the role of postoperative rehabilitation in improving patients' mobility. Periodic QoL screening should be done as early as possible to detect any further decrease. Future research should standardize postoperative interview intervals to improve QoL evaluation and include a control group.

## Introduction

Hip fractures are common and significant injuries that are becoming more prevalent as the population ages [1,2]. They are estimated to increase worldwide in the coming years to 3.94 million cases by 2025 and 6.26 million by 2050, with 4.87% and 6.69% of these cases expected in the Middle East, respectively [1]. This expected rise is alarming, especially when considering the high mortality rate after hip fractures in the first month (8-10%) and in the first year (20-28%) [3-9]. Moreover, these injuries have evolved into a unique worldwide health problem with considerable socioeconomic repercussions for patients and their families, as well as the healthcare systems. It was shown that geriatric patients with hip fractures who undergo surgical procedures are more likely to develop transient mental disorders, depression, and dementia [10]. Nevertheless, the financial impact of hip fractures cannot be overlooked, as the overall one-year healthcare direct cost of hip fractures ranged from 52,232 Canadian dollars for females to 54,289 Canadian dollars for males in Canada [11].

Hip fractures significantly impact a patient's health status and quality of life. Several factors tend to affect the postoperative quality of life, such as mental state, gender, the presence of other comorbidities, nutrition, hospital stay duration, postoperative complications, and the type of surgery [12]. In addition, family and social support play a role in influencing the perceived quality of life [13]. Moreover, those patients are prone to life-threatening conditions, including pneumonia, deep venous thrombosis, urinary retention, pressure ulcers, and cardiac problems [14]. As a result, restoring them to pre-fracture health status is considered a challenge, imposing a significant burden on their caregivers [12,15].

The purpose of this study is to assess the quality of life in patients who underwent hip fracture surgery. We hypothesize that the quality of life is low in elderly patients after hip fracture surgery. According to our search, no studies have tackled this issue in our country, making this the first study to provide guidance for all orthopedic surgeons dealing with similar situations, thus improving orthopedic care.

## Materials and methods

Study design and population

In this is a cross-sectional study, clinical notes for all 550 patients who were admitted to Jordan University Hospital after sustaining a hip fracture and were treated surgically regardless of the type of surgery in the period between February 2016 and December 2019 were examined. After excluding patients who had hip fractures other than femur neck or intertrochanteric fractures, patients who were dead at the time of data collection, and patients who did not want to participate, 199 patients were included in this study. No specific age or gender was required for the inclusion.

In addressing femur neck fractures, our treatment approach involved the use of hemiarthroplasty for elderly patients, while fixation was implemented for individuals under the age of 65. The choice between cannulated screws, sliding hip screws, or a femoral neck system for fixation was determined by the surgeon's preference and the availability of the implant during surgery. In cases where a displaced fracture prevented successful reduction, a total hip replacement was undertaken. Regarding intertrochanteric fractures, our strategy encompassed the use of sliding hip screws or proximal femoral nails, selected based on fracture stability (fracture geometry) and the surgeon's preference. This comprehensive approach aimed to tailor the treatment to the specific needs of each patient, considering factors such as age, fracture type, and surgical considerations.

Data collection tool and process

The participants completed the EuroQol 5-Dimensions 5-Levels (EQ-5D-5L) questionnaire and the EuroQol Visual Analog Scale (EQ-VAS), through face-to-face and telephone-based interviews, in the period between November 8, 2021, and January 5, 2022. Before conducting the cross-sectional study, we established standardized procedures for administering the interviews and rating the quality of life (QoL) assessments to ensure consistency among interviewers. Moreover, participants with cognitive impairments had a family member explain the question to them or provide an answer if they knew it aimed to minimize potential response biases and enhance the reliability of responses.

The Arabic version of EQ-5D-5L was used, which has been shown to be valid and reliable in measuring the quality of life among the our population [16]. In addition, the data on age, gender and type of fracture were collected. The EQ-5D-5L questionnaire is a validated survey that assists only with the basics of daily life activities. It is designed and certified by the Euro Quality of Life (EuroQol) group foundation. The EQ-5D-5L consists of five dimensions. Each dimension represents a Likert scale question with five levels: no problems (level 1), slight problems (level 2), moderate problems (level 3), severe problems (level 4), and extreme problems (level 5). The EQ-VAS is a rating scale question that displays the participant’s self-rated health on a 20 cm vertical, visual analogue scale, which ranged from 0 to 100, where 0 represents "the worst health you can imagine" and 100 represents "the best health you can imagine".

The five dimensions in the EQ-5D-5L questionnaire are about mobility, self-care, usual activities (e.g., work, study, housework, family or leisure activities), pain/discomfort, anxiety/depression. The EQ-VAS was a scale to describe the health situation on the day of collecting data. The internal consistency was examined by calculating Cronbach's alpha for the five measurements of the EQ-5D-5L scale, and the values were found to be 0.782.

Statistical analysis

The data were analyzed using IBM SPSS software version 28.0. (IBM, Inc., Armonk, US). We used mean±SD to describe continuous variables (e.g., age, EQ-VAS score), frequencies and percentages to summarize other categorical data (e.g., gender). The reliability of the EQ-5D-5L scale was computed using Cronbach's alpha. The Pearson's chi-squared test and Fisher-Freeman-Halton test were used to analyze the relationship between the gender and type of fracture with the dimensions of EQ-5D-5L. An independent sample t-test was used to analyze the mean difference between gender and type of fracture with EQ-VAS score and data is presented as mean±SD. Pearson correlation coefficient (r) was calculated to detect relationships between age and EQ-VAS score. A p-value of <0.05 was considered statistically significant.

## Results

A total of 199 patients met our inclusion criteria and agreed to participate in our study. The mean age of the study participants was 75.45±9.0 and ranged from 50 to 105 years. There were 103 males (51.8%) and 96 females (48.2%). As seen in Table 1, 103 (51.8%) patients had femur neck fractures, and 96 (48.2%) patients had intertrochanteric fractures.

**Table 1:** Demographic information of the study sample

Categories	Variables	n (%)
Gender	Male	103 (51.8)
	Female	96 (48.2)
Age (years)	<70	48 (24.1)
	70-90	146 (73.4)
	>90	5 (2.5)
Type of fracture	Femur neck fracture	103 (51.8)
	Intertrochanteric fracture	96 (48.2)

Participants had the highest frequencies of reported problems in performing usual activities (84.4%), followed by mobility (78.9%), self-care (76.9%), pain/discomfort (58.8%), and anxiety/depression (40.2%). Upon analysis, there was a statistically significant association between increased age and having problems with mobility (p=0.023), self-care (p<0.001), and usual activities (p=0.029). The relationship was not significant when it came to pain/discomfort (p=0.95) or anxiety/depression (p=0.28). Table 2 demonstrates the frequencies of reported problems by age group.

**Table 2:** Frequencies and percentages of reported problems for the participants by EQ-5D-5L dimension and age group EQ-5D-5L - EuroQol 5-Dimensions 5-Levels

EQ-5D-5L dimensions		Age groups			p-value
		<70 years	70-90 years	>90 years	
Mobility	No problems	16 (33.3)	24 (16.4)	2 (40)	0.023
	Problems	32 (66.7)	122 (83.6)	3 (60)	
Self-care	No problems	21 (43.8)	25 (17.1)	0 (0)	<0.001
	Problems	27 (56.3)	121 (82.9)	5 (100)	
Usual activities	No problems	13 (27.1)	17 (11.6)	1 (20)	0.029
	Problems	35 (72.9)	129 (88.4)	4 (80)	
Pain/discomfort	No problems	19 (39.6)	61 (41.8)	2 (40)	0.95
	Problems	29 (60.4)	85 (58.2)	3 (60)	
Anxiety/depression	No problems	33 (68.8)	84 (57.5)	2 (40)	0.28
	Problems	15 (31.3)	62 (42.5)	3 (60)	

We also found a statistically significant association between gender and having problems with self-care, as males were more likely to report having problems with self-care compared to females (OR: 3.63; 95% CI: 1.77-7.44; p<0.001). Table 3 summarizes the frequencies of reported problems by gender. Moreover, there was no significant association between the type of fracture and reported problems in any dimension of the EQ-5D-5L questionnaire (p>0.05), as shown in Table 4.

**Table 3:** Frequencies and percentages of reported problems for the participants by EQ-5D-5L dimension and gender EQ-5D-5L - EuroQol 5-Dimensions 5-Levels

EQ-5D-5L dimensions		Gender		p-value
		Male	Female	
Mobility	No problems	20 (19.4)	22 (22.9)	0.55
	Problems	83 (80.6)	74 (77.1)	
Self-care	No problems	13 (12.6)	33 (34.4)	<0.001
	Problems	90 (87.4)	63 (65.6)	
Usual activities	No problems	14 (13.6)	17 (17.7)	0.42
	Problems	89 (86.4)	79 (82.3)	
Pain/discomfort	No problems	37 (35.9)	45 (46.9)	0.12
	Problems	66 (64.1)	51 (53.1)	
Anxiety/depression	No problems	59 (57.3)	60 (62.5)	0.45
	Problems	44 (42.7)	36 (37.5)	

**Table 4:** Frequencies and percentages of reported problems for the participants by EQ-5D-5L dimensions and type of fracture EQ-5D-5L - EuroQol 5-Dimensions 5-Levels

EQ-5D-5L dimensions		Type of fracture		p-value
		Femur neck	Intertrochanteric	
Mobility	No problem	24 (23.3)	18 (18.8)	0.43
	Problem	79 (76.7)	78 (81.3)	
Self-care	No problem	26 (25.2)	20 (20.8)	0.46
	Problem	77 (74.8)	76 (79.2)	
Usual activities	No problem	14 (13.6)	17 (17.7)	0.42
	Problem	89 (86.4)	79 (82.3)	
Pain/discomfort	No problem	42 (40.8)	40 (41.7)	0.9
	Problem	61 (59.2)	56 (58.3)	
Anxiety/depression	No problem	68 (66)	51 (53.1)	0.064
	Problem	35 (34)	45 (46.9)	

The overall mean EQ-VAS score for the participants was 61.46±18.46. There was no significant difference between the mean EQ-VAS score and gender (p=0.092) or type of fracture (p=0.28). Table 5 shows the difference in the mean values of the EQ-VAS score between males and females according to the type of fracture. Increased age was significantly associated with decreased EQ-VAS scores (r=-0.213, p=0.003).

**Table 5:** The mean of the EQ-VAS score among males and females and according to the type of fracture EQ-VAS - EuroQol Visual Analog Scale

Categories	Variables	EQ-VAS score (mean±SD)	p-value
Gender	Male	59.33±19.44	0.092
	Female	63.75 ±17.16	
Type of fracture	Femur neck fracture	62.84±16.6	0.28
	Intertrochanteric fracture	59.98±20.25	

## Discussion

This study aimed to determine the impact of hip fractures on the quality of life of patients who underwent surgery. Our study demonstrated that respondents had difficulties executing their usual activities, mobility, and self-care, as well as reporting pain and anxiety. Age negatively impacted the ability to move, perform usual activities, and self-care. In addition, males were more likely to experience problems with self-care. However, no association was seen between the mean EQ-VAS score and gender or the fracture type. 

When taking into consideration the projected increase in the prevalence of hip fractures in the upcoming years [17], there is a need to predict the possible outcomes regarding the quality of life of patients surgically treated for hip fractures, as it may impose a great burden on the patients themselves and their families, especially with the difficult economic status in our country. In order to appropriately study those outcomes, an accurately designed assessment tool is required. In this area, there are various frequently used instruments, such as the EQ-5D-5L survey and the Hip Disability and Osteoarthritis Outcome Score (HOOS) [18,19]. Our results demonstrate a significant relationship between undergoing hip fracture surgery and patients' mobility. In other words, the ability of surgically treated patients to move declines, especially with increased age. This observation aligns with the study conducted by Amarilla-Donoso et al., which tackled the quality of life after hip fractures in the elderly [13]. In addition, our observation confirms that of Gjertsen et al., who highlighted the decline in mobility with increasing age in patients undergoing femur neck fracture surgeries in Norway [20].

Femoral neck fractures and intertrochanteric fractures are considered the most prevalent kinds of osteoporosis-related hip fragility fractures [21]. These individuals require a special rehabilitation plan in order to return to their pre-fracture status. In our study, more than three-quarters of the participants reported problems with self-care. This outcome is consistent with what other studies have found [20,22]. However, our results showed that males were more likely to report self-care difficulties than females. In this context, a study conducted by Amarilla-Donoso et al. with a sample of 76.3% females has concluded that patients, in general, experience considerable deterioration in their self-care as a result of hip fracture surgery [13]. From our perspective, these conflicting results could have a social dimension. In other words, females in some societies like ours might have been reluctant to report any decline in their self-care status as it may have negatively impacted their self-image. 

Our results demonstrated that performing usual activities significantly decreases with age. This can be explained by the fact that the body's capacity to tolerate and recover from stressors, such as hip fractures, is impeded by the drop in physiological reserves that is brought on by aging [23]. This can be referred to as frailty, which is characterized by a loss of lean body mass, strength, endurance, balance, walking performance, and low activity [24,25]. In addition, no significant decline was seen in the domains of pain and anxiety in relation to aging. This contrasts with what Gjertsen et al. have found [20]. We hypothesize that this discrepancy in the outcomes is due to inadequate postoperative follow-up as our country lacks a national-computerized patient record system that connects different healthcare facilities [26].

It is worth noting that our study did not consider the type of surgery performed or pre-existing comorbidities as variables in assessing the quality of life after hip fracture surgery. Instead, our study has considered the type of fracture as a variable potentially impacting the quality of life of hip fracture patients. Nevertheless, our results have shown that the fracture type did not significantly affect the quality of life after the surgeries. In this regard, the study conducted by Gjertsen et al. considered the type of surgery performed to assess the health-related quality of life in hip fracture patients, comparing internal fixation surgeries and hemiarthroplasty [27]. Their study showed that patients treated with bipolar hemiarthroplasty had less pain and more satisfaction with the results of the operation and also found a marked reduction in EQ-5D-5L index score for both target groups, with the bipolar hemiarthroplasty having a better EQ-5D-5L index score than internal fixation [27]. Furthermore, Buecking et al. considered pre-existing comorbidities to study factors that affect the health-related quality of life in patients with femur neck fracture; they found that there are pre-existing factors that may influence the outcome of the surgery, such as depression and cognitive impairment [28].

The findings of this study must be interpreted with caution due to several limitations. Firstly, the sample size was relatively small compared to other studies [20,27]. Secondly, the lack of standardization in the interview time following hip fracture surgery could potentially impact the participants' quality of life scores. Thirdly, the cross-sectional design of the study increases the risk of recall bias. Finally, our study did not consider other comorbidities that might influence patients' quality of life. However, one of the study's strengths is that it is the first study among our population to assess patients' quality of life following hip surgery comprehensively. Moreover, the study used the EQ-5D-5L survey, which is a reliable tool for assessing the quality of life of patients undergoing hip fracture surgery. Therefore, we believe that our results demonstrated significant findings that may serve as the basis for further research. We suggest that future studies establish standardized intervals for postoperative interviews to ensure a more consistent assessment of QoL outcomes, as well as to reinvestigate after rehabilitation and have a control group to compare with.

## Conclusions

In conclusion, our results showed that hip fracture surgeries were significantly related to lower mobility, self-care, and usual activities, which was more apparent in higher age groups. This emphasizes the need to increase the awareness of patients regarding the possible decline in their quality of life and the role of postoperative rehabilitation in trying to improve patients' mobility as much as possible. Moreover, we suggest that screening tools for quality of life should be used regularly to detect any further decline early, which will be beneficial for the patient and the healthcare system.
